# Management of rotator cuff injuries using allogenic platelet-rich plasma

**DOI:** 10.1186/s13018-024-04657-4

**Published:** 2024-03-04

**Authors:** Ashim Gupta, Filippo Migliorini, Nicola Maffulli

**Affiliations:** 1 Regenerative Orthopaedics, Noida, 201301 India; 2 Future Biologics, Lawrenceville, GA 30043 USA; 3 South Texas Orthopaedic Research Institute (STORI Inc.), Laredo, TX 78045 USA; 4 BioIntegrate, Lawrenceville, GA 30043 USA; 5 Department of Orthopaedic, Trauma, and Reconstructive Surgery, RWTH University Medical Centre, 52074 Aachen, Germany; 6 Department of Orthopedics and Trauma Surgery, Academic Hospital of Bolzano (SABES-ASDAA), Teaching Hospital of the Paracelsus Medical University, 39100 Bolzano, Italy; 7 Department of Trauma and Orthopaedic Surgery, Faculty of Medicine and Psychology, University La Sapienza, Rome, Italy; 8 Barts and the London School of Medicine and Dentistry, Centre for Sports and Exercise Medicine, Queen Mary University of London, London, E1 4DG UK; 9 School of Pharmacy and Bioengineering, Keele University School of Medicine, Stoke on Trent, ST5 5BG UK

**Keywords:** Platelet-rich plasma, PRP, Allogenic PRP, Regenerative medicine, Rotator cuff injuries, Rotator cuff tear, Subacromial impingement syndrome

## Abstract

Rotator cuff injuries are a major cause of shoulder pain, affecting the quality of life and producing a significant burden on healthcare systems. Conservative management modalities are prioritized, resorting to surgery only when required. The field of regenerative medicine involving the use of biologics, such as platelet-rich plasma (PRP), has evolved and shown potential for managing rotator cuff injuries. Nonetheless, limitations including subpar outcomes have led clinicians to question the efficacy of autologous PRP. To circumvent this, the possibility of utilizing a standardized and well-characterized allogenic PRP for RCI has been explored. In this manuscript, we qualitatively present the evidence from in vitro, pre-clinical, clinical and ongoing studies investigating the applications of allogenic PRP in the context of rotator cuff disorders. Administration of allogenic PRP is safe and potentially efficacious to manage rotator cuff injuries, though more adequately powered randomized controlled trials with longer follow-ups are warranted to further establish the efficacy of allogenic PRP and justify its routine clinical use.

## Introduction

Musculoskeletal injuries affect billions of people worldwide, and markedly impact their quality of life [1]. Shoulder pain is the third foremost musculoskeletal condition, and its occurrence increases over age, with a lifetime incidence of about 70%, and is highest in 40–59-year-old individuals, leading to a substantial socio-economic burden [2, 3]. Rotator cuff injuries (RCI) are the most frequent cause of shoulder pain, accounting for over 70% of such complaints [4, 5]. RCI include a continuum of disorders, such as partial or full-thickness tears, subacromial impingement syndrome, and cuff tear arthropathy [6]. Conservative management modalities to manage RCI include the use of pharmacological agents such as non-steroidal anti-inflammatory drugs, corticosteroids, and opioids; non-pharmacological options such as physical therapy, activity modification, acupuncture, and electrotherapies; and surgical intervention after conventional therapies have failed [7]. These traditional options have flaws and side effects, and typically provide only short-term symptomatic relief instead of addressing the underlying pathologies associated with RCI [8].

There has been considerable interest in the use of biologics, including platelet-rich plasma (PRP) for musculoskeletal regenerative medicine applications [9]. Several studies, including systematic reviews and meta-analyses, randomized controlled trials, case studies and reports, etc. have shown the safety and efficacy of PRP [9]. In contrast, the subpar outcomes associated with a lack of standardization and characterization of PRP preparations, and patient-related variables including age, comorbidities, mental and physical stress levels, alcohol consumption, smoking status, concomitant medication, etc. have led to questioning its efficacy [9, 10]. To circumvent the limitations presented by autologous PRP, the possibility of using well-characterized allogenic PRP formulation with a standardized preparation protocol has been explored in patients with RCI. The primary objective of this study is to report the in vitro, preclinical, and clinical outcomes of allogenic PRP for the management of RCI. The secondary objective is to document the ongoing clinical trials registered on various trial protocol repositories associated with allogenic PRP for the management of RCI.

## Materials and methods

### Search criteria

**Fig. 1:**
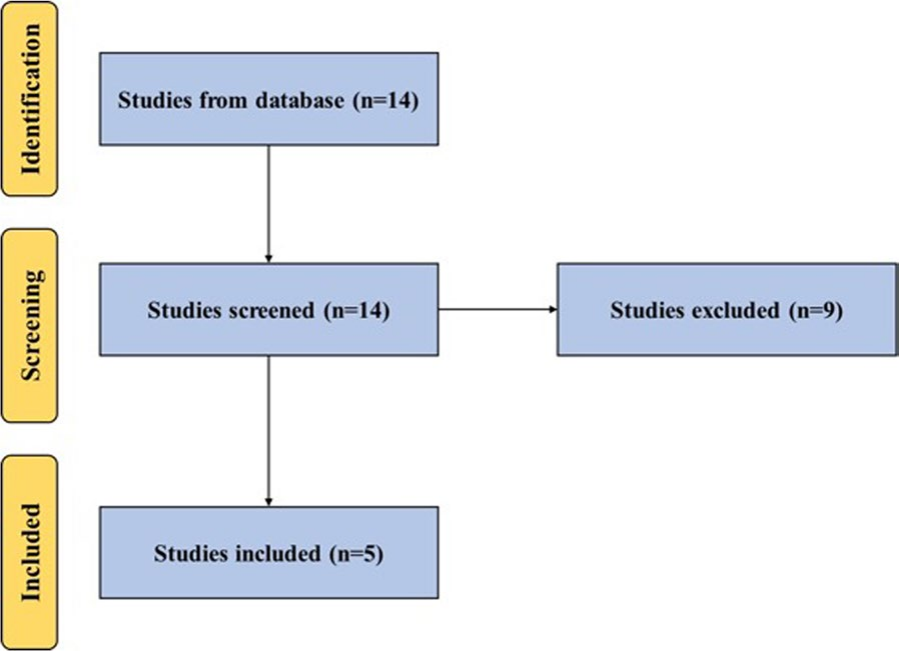
A PRISMA flow diagram outlining the record identification and selection process

A systematic search on PubMed (MEDLINE) and Scopus was conducted, aiming to retrieve relevant articles published in English until September 2023. Adherence to the PRISMA statement and guidelines was maintained throughout the study, using the designated search terms: (allogenic platelet-rich plasma' OR 'allogenic PRP') AND (‘shoulder’ OR 'rotator-cuff' OR 'rotator cuff injuries' OR ‘subacromial impingement syndrome’). All studies utilizing allogenic PRP for RCI were included. Studies not using allogenic PRP or not focusing on the management of RCI were excluded. Figure 1 illustrates the systematic search performed.

In addition, we searched ClinicalTrials.gov, Chinese Clinical Trial Register (ChiCTR) and Clinical Trials Registry—India (CTRI) using the aforementioned search terms to identify registered trials on the use of allogenic PRP for the management of RCI.

## Results

### In vitro studies

Hilber et al. [11] investigated the effect of allogenic leukocyte-poor PRP on human tenocytes post-treatment with prednisolone and assessed standardization for its application for clinical use. Venous blood was collected from healthy volunteers (n = 30) who had not used any medication for at least 2 weeks to prepare leukocyte-poor PRP using an Arthrex double syringe system. Tenocytes were isolated from discarded rotator cuff segments from 6 patients. The cells were divided into 5 groups to analyse cell cycle kinetics to assess proliferation. All groups were first treated with 40 mg/mL methylprednisolone for 1 h. The control group was then kept in 2% FCS for 72 h. The first treatment group was kept in 2% FCS for 48 h followed by incubation with PRP (10 and 20% PRP) for 24 h. The second treatment group was kept in 2% FCS for 24 h followed by incubation with PRP (10 and 20% PRP) for 48 h. In the remaining two groups, the effect of leukocyte-poor PRP (10 and 20% PRP) after 48 and 72 h of incubation compared to 10% FCS alone was determined. All experiments were performed using tenocytes from 3 donors within 2 passages. The results demonstrated a significant increase in tenocyte proliferation post-treatment with PRP (both 10 and 20%) compared to FCS alone at 48 h, but no differences were reported at 72 h. In the first treatment group, no differences were observed between the PRP (either 10 or 20% PRP) and the control group. On the other hand, in the second treatment group, a significant increase in tenocyte proliferation post-treatment with PRP (both 10 and 20%) compared to control was observed. No differences were observed between 10 and 20% PRP among any group. The main shortcomings of this study were the small sample size for tenocyte isolation and the lack of characterization data, i.e., the number of platelets, leukocytes and red blood cells (RBCs) in the PRP formulation used. Nevertheless, allogenic leukocyte-poor PRP increases the tenocyte proliferation and antagonizes the harmful impact of prednisolone 24 h post-treatment.

Jo et al. [12] assessed the effect of allogenic PRP on tenocytes with or without interleukin-1β (IL-1β) induced inflammation. Tenocytes were enzymatically isolated and cultured from patients with RC tear. PRP was prepared using a plateletpheresis system with a leukoreduction set. Platelet count was concentrated to 4000 × 10^3^ platelets/µL (PRP4000) and then diluted to 1000 × 10^3^ platelets/µL (PRP1000) and 200 × 10^3^ platelets/µL (PRP200). 10% calcium gluconate was used as an activator. The tenocytes were allowed to attach for 24 h and were then treated with 1 ng/mL of IL-1β, 10%vol/vol platelet-poor plasma (PPP) and 10% vol/vol PRPs for 24 h. Untreated tenocytes were used as a control. Gene expression analysis via Real-time Reverse Transcriptase Polymerase Chain Reaction (RT-PCR) for pro-inflammatory cytokines, degradative enzymes and their inhibitors, anti-inflammatory cytokines and matrix molecules was performed. Protein synthesis analysis was also performed via Western blotting. Afterwards, a cell viability assay utilizing an EZ-CyTox cell viability assay kit was performed. The characteristics for PPP were 3.00 ± 1.41 × 10^6^ platelets/µL, no RBCs/µL and 0.01 ± 0.01 × 10^6^ white blood cells (WBCs)/µL; PRP200 were 197.50 ± 9.19 × 10^6^ platelets/µL, 0.04 ± 0.00 × 10^6^ RBCs/µL and 0.01 ± 0.01 × 10^6^ WBCs/µL; PRP1000 were 850.00 ± 67.88 × 10^6^ platelets/µL, 0.18 ± 0.01 × 10^6^ RBCs/µL and 0.01 ± 0.02 × 10^6^ WBCs/µL; and PRP4000 were 3372.50 ± 412.24 × 10^6^ platelets/µL, 0.68 ± 0.17 × 10^6^ RBCs/µL and 0.01 ± 0.01 × 10^6^ WBCs/µL. Without IL-1β treatment, PRP1000 and PRP4000 significantly induced the expression of pro-inflammatory cytokines *IL-1β*, *IL-6*, *COX-2* and *mPGES-1*; they inhibited the expression of *TNF-α*. PPP, PRP1000 and PRP4000 induced the expression of matrix degradative enzymes and inhibitors *MMP-1*, *MMP-3*, *MMP-13*, *ADAMTS-4* and *TIMP-3,* while inhibiting the expression of *ADAMTS-5*. PPP and PRPs decreased the expression of anti-inflammatory cytokines *IL-4* and *VIP*; while PRPs increased the expression of *IL-1RA*. PPP and PRPs decreased the expression of *collagen type I and III* and *decorin*; while increasing the ratio of collagen type I: III. With IL-1β treatment, IL-1β significantly upregulated the expression of pro-inflammatory cytokines *IL-1β*, *IL-6*, *COX-2* and *mPGES-1*. Treatment with PRP4000 significantly downregulated the expression of *IL-1β* and *mPGES-1*; PPP and PRPs downregulated the expression of *IL-6*; and PPP and PRP200 downregulated the expression of *COX-2*. IL-1β also significantly upregulated the expression of *MMP-1*, *MMP-3*, *MMP-9*, *MMP-13* and *ADAMTs-4*. No significant differences were observed for these post-treatments with PPP and PRPs. With IL-1β treatment, IL-1β significantly inhibited the expression of anti-inflammatory cytokines *IL-4* and *VIP* and induced the expression of *IL-10* and *IL-1RA*. PPP and PRP1000 increased the expression of *IL-4*, PRP1000 and PRP4000 increased the expression of *VIP*, and PPP and PRPs significantly decreased the expression of *IL-10* and *IL-1RA*. With IL-1β treatment, *IL-1β* significantly downregulated the expression of *collagen type I* and upregulated the expression of *collagen type III*. Treatment with PRP200, PRP1000 and PRP4000 decreased the expression of *collagen type III*. For protein synthesis, without IL-1β treatment, PPP and PRPs induced the synthesis of MMP-3, PRPs induced synthesis of TIMP-1, and PRP1000 and PRP4000 induced synthesis of TIMP-3. On the other hand, IL-1β promoted the synthesis of MMP-13. Treatment of PPP and PRPs decreased the synthesis of MMP-1 and PRP200 decreased the synthesis of MMP-3. Lastly, either with or without IL-1β, PPP and PRPs increased tenocyte proliferation in a dose-dependent manner. The results from this study demonstrated the pleiotropic effect of PRP on tenocytes subject to the presence of inflammation, and PRP induced inflammation in the absence of inflammation and ameliorated it in the presence of inflammation.

### Pre-clinical studies

To date, there are no published preclinical studies involving the use of allogenic PRP for the treatment of RCI.

### Clinical studies

Jo et al. [13] in a retrospective cohort study investigated the safety and efficacy of allogenic PRP compared to autologous PRP in arthroscopic RC tear. Patients with full-thickness RC tear treated arthroscopically with allogenic PRP and with a minimum follow-up of 24 months were included in the study. 10 patients in the allogenic PRP and 7 patients in the autologous PRP group were enrolled. PRP was formulated utilizing a plateletpheresis system with a leukoreduction set. The platelet count was adjusted to 1000 × 10^3^ platelets/µL, and a gel was formed by using 10% calcium gluconate in 3 mL of PRP. Three PRP gels (3 mL each) threaded to a suture were inserted between the torn end and the greater tuberosity. Clinical outcomes were measured as pain, range of motion (ROM), muscle strength, overall satisfaction and functional scores. The structural integrity was measured using Sugaya’s magnetic resonance imaging (MRI) classification for patients. The change in the cross-sectional area of the supraspinatus was also measured. No adverse effects were observed for allogenic PRP throughout the study. Both groups showed a significant reduction in VAS scores compared to baseline, but there was no statistically significant difference between the groups. Forward flexion, abduction, and internal rotation significantly improved in the allogenic PRP group, while no difference was observed in external rotation with the arm at the side. In contrast, for the autologous PRP group, there was significant improvement in external rotation with the arm at the side, but no improvements were observed for forward flexion, abduction and internal rotation. No differences between the two groups were observed for ROM. Supraspinatus and subscapularis strength showed significant improvements for the allogenic PRP group, but no differences were observed for the autologous PPR group. No changes were observed for infraspinatus strength in either group. No differences were observed between the groups for either supraspinatus, infraspinatus or subscapularis strength. Patients in both groups showed overall satisfaction, with no difference between the groups. Both groups demonstrated improvement in overall function. ASES, Constant, DASH, SST and SPADI scores also improved significantly for both groups, with no differences between the groups. In addition, no significant differences in the retear rate and cross-sectional area of the supraspinatus were observed between the two groups. The administration of allogeneic PRP is safe, and efficacy concerning clinical and structural outcomes is similar to autologous PRP. This study has several limitations, including small sample size, heterogeneity in participants (some patients in the autologous PRP group had partial tears), lack of randomization, and incomplete characterization of PRP formulations. Despite these, this is one of the first studies to evaluate the effectiveness of allogeneic PRP compared to autologous PRP in patients with RCI.

Jo et al. [12], in another retrospective study, evaluated the safety and efficacy of allogenic PRP in patients with RCI in comparison with propensity score-matched control patients treated with corticosteroids. The inclusion criteria included patients with RC disease treated with allogenic PRP at the authors’ institution. The exclusion criteria included patients with bilateral shoulder pain, presence of partial or full thickness RC tears, cuff arthropathy, restriction of both active and passive ROM of the shoulder joint of 25% in at least two directions, prior subacromial injection in the last 3 months, history of cancer, surgery, trauma, symptomatic spinal disorders, diabetes and lack of follow-up data at 6 months. 17 patients met the inclusion/exclusion criteria and were enrolled in the PRP group. All injections were administered with the patient seated with the arm rotated internally in front of the abdomen under ultrasonography. Either 4 mL of allogenic PRP1000 (characterization described above) or propensity-score matched controls, 1 mL of triamcinolone acetonide (40 mg/mL) in 3 mL saline, were injected in the subacromial space. All pain medications except the rescue analgesic (37.5 mg tramadol + 375 mg acetaminophen) were discontinued. Outcome measures were recorded at baseline and 1 week, 1 month, 3 months and 6 months post-injection. Clinical outcome measures included pain (via VAS), ROM (active forward flexion, abduction, external rotation with the arm at the side, and internal rotation using vertebral levels), muscle strength (supraspinatus, infraspinatus, and subscapularis muscles with an electronic scale), functional scores (ASES score, the constant score, UCLA score, DASH questionnaire, SST and SPADI), and overall satisfaction and function (via SANE). No adverse events were reported with allogenic PRP throughout the study. The allogeneic PRP group showed a significant reduction in pain at 6 months follow-up compared to baseline. In contrast, the steroid group showed a significant reduction in VAS at 1-month follow-up compared to baseline, but the pain increased at 3- and 6-month follow-up. No changes were observed for ROM for either group compared to baseline or between the groups. The allogeneic PRP group also showed significant improvement in the strength of the three muscles at 3 and 6 months, whereas in the steroid group, only a temporary improvement was observed in the infraspinatus and subscapularis muscles at 1 month. Additionally, the strength of the supraspinatus in the allogenic PRP groups was higher than in the steroid group at 3 and 6 months, and for the infraspinatus muscle, it was higher at 3 months. No differences were reported for subscapularis muscles between the two groups. Moreover, all functional scores showed improvement for the allogenic PRP group compared to baseline at 6 months. Interestingly, similar to pain scores, in the steroid group, the functional scores improved quickly at 1 month compared to the PRP group but deteriorated at later follow-ups. Lastly, the overall satisfaction and function improved significantly for the allogenic PRP group, while no improvement was observed for the steroid group. The main shortcomings of this study are its small size and the lack of randomization. Nevertheless, this study demonstrated that allogenic PRP was superior to corticosteroids in terms of reducing pain and improving function in patients with RCI in the longterm.

As a follow-up to the aforementioned retrospective study [12], Jo et al. [14] conducted a randomized controlled trial to assess the safety and efficacy of a subacromial injection of allogenic PRP compared to corticosteroid in patients with RCI. The inclusion criteria included patients ≥ 18 years of age, with unilateral shoulder pain for at least 3 months, who presented with either a Neer or Hawkins impingement sign, either a painful arc or a positive Jobe test. The exclusion criteria included subacromial injections in the last 3 months, history of shoulder trauma, full-thickness RC tear and restriction of both active and passive ROM of the glenohumeral joint of 25% in at least 2 directions compared to contralateral shoulder or normal values. Patients were randomized 1:1, with block sizes of 4 and 6 to the allogenic PRP or corticosteroid group. PRP was prepared as described earlier [12] and consisted of 988.67 ± 60.10 platelets/mL. Either 4 mL of allogenic PRP or a mixture of 1 mL of 40 mg/mL triamcinolone acetonide and 3 mL of 2% lidocaine was administered under ultrasonographic guidance. Outcome measures were recorded at baseline and 1 week, 1 month, 3 months and 6 months follow-up. The primary outcome measure was safety and Constant Score at 1 month. The secondary outcomes included pain, ROM, muscle strength, function (SPADI, ASES, UCLA, SST and DASH questionnaires), and overall satisfaction and function. No adverse events related to allogenic PRP or steroids were reported during the study. The Constant Score for the allogenic PRP improved over time, and was significantly higher at 6 months compared to baseline. On the other hand, for the steroid group, the Constant Score was significantly higher at 1 month but worsened towards baseline at later follow-ups. A similar outcome was observed for pain scores, functional scores and overall satisfaction. For ROM, a significant improvement was observed for external rotation with the arm at the side in the allogenic PRP group. No differences in strength in either group compared to baseline were observed. No differences were observed between the groups except for overall satisfaction. This study has several limitations, including lack of a placebo control group, inadequate follow-up duration, no assessment of structural changes in subacromial space, and utilization of pure PRP (i.e., no leukocytes or RBCs were present which may alter the outcomes). Yet, this study was one of the first randomized controlled trials utilizing allogenic PRP for RCI, demonstrating that administration of allogenic PRP is safe, and can reduce pain and improve function over the long term compared to corticosteroids, which are quick-acting and effective for short-term only.

El-Sherif et al. [15] investigated the efficacy of platelet-derived lyophilized growth factors (L-GFs) compared to a placebo in a double-blind, placebo-controlled trial for the management of subacromial impingement syndrome. The inclusion criteria included all patients with subacromial impingement syndrome at the authors’ institution who did not respond to conservative treatment for 3 months. Exclusion criteria included patients with a history of shoulder surgery, fracture, dislocation or subluxation, full-thickness RC tear, positive “drop arm sign”, degenerative arthropathy or frozen shoulder of the glenohumeral joint, upper extremity or cervical spine disorders with a noteworthy impact on the shoulder, diabetes mellitus, active infection or other painful, function-limiting disorders of the shoulder and significant systemic disease. 60 patients were enrolled in this study and randomized equally in two groups. Groups included ultrasound-guided single subacromial injection of either saline or L-GFs. L-GFs were supplied as a powder, with a growth factor concentration equivalent to those obtained from 10^6^ platelets/µL from 20 mL blood. Patients were assessed at baseline for tender points, provocative tests such as painful arc, Neer’s sign, Hawkins-Kennedy and empty can test, active and passive ROM, muscle power around the shoulder, VAS and SPADI. No adverse events were reported during the study. At follow-up at 8 weeks, patients were reassessed using ultrasound guidance, and VAS and SPADI. ROM showed a statistically significant improvement in passive flexion and abduction in both groups at 8 weeks compared to baseline, and a significantly higher abduction in the L-GFs group. Active flexion and active and passive internal rotation and extension showed significant improvement in the L-GFs group only. Both groups also showed significant improvement in VAS and SPADI (both pain and total mean values) at 8 weeks, and the improvement in the L-GFs group was statistically better compared to the saline group. In addition, the L-GFs group showed significant improvement in the SPADI disability scale at 8 weeks follow-up. Additionally, only subacromial tenderness and painful arc improved significantly in the L-GFs group at 8 weeks follow-up, and no differences were observed for other provocative tests. Ultrasonographic analysis showed significant improvement in supraspinatus tendon thickness only at 8 weeks follow-up for the L-GFs group. In summary, administration of the L-GFs is safe, with significant improvement in pain and functional disability outcome measures in SIS patients. Moreover, a significant reduction in supraspinatus tendon thickness can potentially lead to proper healing and function in patients with SIS.

### Ongoing clinical studies

As of September 6, 2023, there are no clinical trials registered on ClinicalTrials.gov, Chinese Clinical Trial Register (ChiCTR) or Clinical Trials Registry—India (CTRI) to study the effects of allogenic PRP for the treatment of RCI.

## Discussions

The present study evaluated the therapeutic potential of allogenic PRP for the management of RCI. In vitro, pre-clinical and clinical studies focusing on the effect of allogenic PRP on RCI were included. Based on our search strategy and inclusion/exclusion criteria, one in vitro study, three clinical studies and one study with both in vitro and clinical study components fit the scope of our manuscript.

RC-related disorders pose a great challenge to clinicians, and the lack of an effective gold standard treatment markedly impacts the quality of life of patients, posing a substantial burden on healthcare systems across the world [1–6]. In an in vitro study, treatment of tenocytes for 48 h, post-incubation with corticosteroids for 24 h, with allogenic PRP increased the rate of tenocyte proliferation [11]. These positive effects were not observed if the tenocytes were treated for 24 h, post-incubation with corticosteroids for a longer duration of 48 h. The beneficial effect of allogenic PRP is restricted to a specific time post-treatment with corticosteroids, something that should be considered in clinical decision-making. Jo et al. [12] attempted to bring clarity to a highly debated topic whereby some researchers reported the anti-inflammatory properties of PRP, whereas others reported catabolic and inflammatory responses, especially in tendon stem cells and fibroblasts post-treatment with PRP. The anti-inflammatory effects of PRP on tenocytes are exerted under inflammatory conditions only, and pro-inflammatory effects are exerted in the absence of inflammation.

Jo et al. [12–14] after an in vitro study, conducted 3 clinical studies including a randomized controlled trial. In the first retrospective cohort study, the administration of allogeneic PRP was safe and led to improvements in pain, ROM, muscle strength, overall satisfaction and functional scores, similar to autologous PRP [13]. In another retrospective study, the administration of allogeneic PRP led to a reduction in pain and improvement in function in the long-term (6 months) compared to baseline. In contrast, the improvement post-injection of corticosteroids was best at 1 month, and it deteriorated at later follow-ups at 3 and 6 months [12]. In a randomized controlled trial, Jo et al. [14] compared the efficacy of allogenic PRP with corticosteroids in patients with RCI. Similar to the retrospective study [12], the administration of allogeneic PRP was safe and it improved pain and function in the longterm compared to the quick, short-term effect of corticosteroids [14]. In another randomized controlled trial, El-Sherif et al. evaluated the efficacy of L-GFs compared to placebo in patients with SIS [15]. The authors reported clinically significant improvements in pain and disability along with a significant decrease in the thickness of the supraspinatus tendon [15]. There are no ongoing clinical trials registered on any clinical trial protocol registries.

## Conclusion

Notwithstanding methodological limitations, in vitro and clinical studies demonstrated that the administration of allogeneic PRP is safe and has similar efficacy to autologous PRP and it can reduce pain and improve function for a longer duration compared to corticosteroids in patients with RC injuries. However, given the dearth of relevant literature, pre-clinical studies to better understand the mechanism of action, and adequately powered, multi-centre, non-randomized and randomized controlled trials with longer follow-up are warranted to further evaluate the efficacy of allogenic PRP in patients with RC injuries.

## Data Availability

The datasets generated during and/or analysed during the current study are available throughout the manuscript.

## References

[1] Aratikatla A, Maffulli N, Rodriguez HC, Gupta M, Potty AG, Gupta A (2022). Allogenic perinatal tissue for musculoskeletal regenerative medicine applications: a systematic review. Biomedicines.

[2] Lin KM, Wang D, Dines JS (2018). Injection therapies for rotator cuff disease. Orthop Clin North Am.

[3] Luime JJ, Koes BW, Hendriksen IJ, Burdorf A, Verhagen AP, Miedema HS, Verhaar JA (2004). Prevalence and incidence of shoulder pain in the general population; a systematic review. Scand J Rheumatol.

[4] Mitchell C, Adebajo A, Hay E, Carr A (2005). Shoulder pain: diagnosis and management in primary care. BMJ.

[5] Östör AJ, Richards CA, Prevost AT, Speed CA, Hazleman BL (2005). Diagnosis and relation to general health of shoulder disorders presenting to primary care. Rheumatology (Oxford).

[6] Jo CH, Shin WH, Park JW, Shin JS, Kim JE (2017). Degree of tendon degeneration and stage of rotator cuff disease. Knee Surg Sports Traumatol Arthrosc.

[7] Hermans J, Luime JJ, Meuffels DE, Reijman M, Simel DL, Bierma-Zeinstra SM (2013). Does this patient with shoulder pain have rotator cuff disease? The rational clinical examination systematic review. JAMA.

[8] Coombes BK, Bisset L, Vicenzino B (2010). Efficacy and safety of corticosteroid injections and other injections for management of tendinopathy: a systematic review of randomised controlled trials. Lancet.

[9] Gupta A, Potty AG, Maffulli N (2023). Allogenic platelet-rich plasma for treatment of knee and hip osteoarthritis. Front Pain Res (Lausanne).

[10] Gupta A, Jeyaraman M, Maffulli N (2022). Common medications which should be stopped prior to platelet-rich plasma injection. Biomedicines.

[11] Hilber F, Loibl M, Lang S, Kerschbaum M, Brockhoff G, Angele P, Zellner J, Schmitz P, Nerlich M, Worlicek M (2017). Leukocyte-reduced platelet-rich plasma increases proliferation of tenocytes treated with prednisolone: a cell cycle analysis. Arch Orthop Trauma Surg.

[12] Jo CH, Lee SY, Yoon KS, Oh S, Shin S (2018). Allogenic pure platelet-rich plasma therapy for rotator cuff disease: a bench and bed study. Am J Sports Med.

[13] Jo CH, Shin JS, Lee SY, Shin S (2017). Allogenic platelet-rich plasma for rotator cuff repair. Acta Ortop Bras.

[14] Jo CH, Lee SY, Yoon KS, Oh S, Shin S (2020). Allogeneic platelet-rich plasma versus corticosteroid injection for the treatment of rotator cuff disease: a randomized controlled trial. J Bone Jt Surg Am.

[15] El-Sherif SM, Abdel-Hamid MM, Noureldin JMAM, Fahmy HM, Abdel-Naby HMA (2023). Effectiveness of lyophilized growth factors injection for subacromial impingement syndrome: a prospective randomized double-blind placebo-controlled study. J Orthop Surg Res.

